# Patient morbidity after impacted third molar extraction with different flap designs. A systematic review and meta-analysis

**DOI:** 10.4317/medoral.23320

**Published:** 2020-02-17

**Authors:** Pablo Glera-Suárez, David Soto-Peñaloza, David Peñarrocha-Oltra, Miguel Peñarrocha-Diago

**Affiliations:** 1 DDS, Master in Oral Surgery and Implant Dentistry, Department of Stomatology, Faculty of Medicine and Dentistry, University of Valencia, Spain; 2 DDS, MSc. Master in Oral Surgery and Implant Dentistry, Department of Stomatology, Faculty of Medicine and Dentistry, University of Valencia, Spain; 3 DDS, PhD. Assistant Professor, Oral Surgery Unit, Department of Stomatology, Faculty of Medicine and Dentistry, University of Valencia, Spain; 4 MD, PhD, DDS. Full Professor, Oral Surgery Unit, Department of Stomatology, Faculty of Medicine and Dentistry, University of Valencia, Spain

## Abstract

**Background:**

To review the literature on the effect of different surgical flaps upon patient morbidity (pain perception, trismus, swelling and osteitis) after impacted third molar extraction.

**Material and Methods:**

An electronic and complementary search of main databases and grey literature was performed up to January 2019 to retrieve randomized clinical trials. The Cochrane risk of bias assessment tool was used for methodological appraisal. A random-effects meta-analysis was conducted of pain perception and trismus.

**Results:**

From the initially 1314 screened studies, only 11 were included in the qualitative synthesis, and 5 in the meta-analysis. There were no statistically significant differences in pain between the envelope and triangular flap designs over time, except on the sixth postoperative day, when the envelope flap proved more painful. Regarding trismus, statistically significant differences were observed on the seventh postoperative day, with greater mouth opening in the envelope flap group than in the triangular flap group. There were no clear differences in swelling and osteitis among the flap designs.

**Conclusions:**

Despite its limitations, the present meta-analysis found no clear differences in patient morbidity between the different flap designs.

** Key words:**Impacted third molar, triangular flap, envelope flap, surgical flap, pain perception, trismus.

## Introduction

Since impacted third molars are not often erupted into the oral cavity, a surgical flap must be raised to remove them, employing ostectomy, tooth sectioning and sutures that may trigger postoperative tissue reactions (1). Several surgical flap designs are reported in the literature, mainly the triangular flap and the envelope flap. Some authors have evaluated these designs in relation to periodontal health outcomes, such as probing depth at the distal surfaces of the adjacent second molar (1).

Other studies have assessed patient post-extraction quality of life (QoL) (2,3). Among the flaps employed for impacted tooth extraction, the standard triangular flap (or three-cornered flap) and the envelope flap are the most widely used designs (1), along with other flap designs. (4-6).

Soft and hard tissue manipulation during third molar extraction may trigger transient effects such as postoperative discomfort, swelling and pain that can adversely affect patient wellbeing (2). Other clinical aspects such as osteitis or infection have also been considered (4,5,7-9). Thus, seeking the best approach to ensure good postoperative quality of life in patients undergoing impacted third molar extraction is an important concern in everyday clinical practice.

Evidence based practice seeks to make informed decisions based on the best available evidence, in confluence with clinical skills and patient preferences (10). However, researchers frequently assess surrogate outcomes (those not related to the patient) instead of patient reported outcomes, which are closely tied to patient preferences and quality of life (11).

A number of studies and reviews have addressed surgical approaches in third molar extraction (7). The present study was therefore carried out to systematically review the literature on the impact of different surgical flap designs upon postoperative pain perception and clinical outcomes after impacted molar extraction.

## Material and Methods

The present systematic review was carried out according to the PRISMA statement (12) and AMSTAR-2 guidelines (13).

- Focused question

A predefined focused question was proposed according to the PICO format (14) as follows: To what extent does flap design during impacted third molar extraction affect postoperative patient morbidity in terms of pain perception and other clinical outcomes?

Population: patients undergoing extraction of an impacted third molar.

Intervention: flap design employed (e.g., envelope flap, szmyd flap, comma-shaped incision).

Comparator: triangular flap.

Outcomes: patient morbidity (pain perception, trismus, swelling and osteitis).

- Electronic search

The electronic search of main databases and grey literature including the Medline via PubMed, EMBASE, Web of Science and OpenGrey (www.opengrey.org) was performed up to February 2019 by two reviewers (PGS and DSP) in duplicate. The medical subject heading (MESH) terms for PubMed, “EMTREE” for Embase and other free-text terms were used and combined whenever possible in each database. Additionally, the reference lists of included studies were consulted to retrieve potentially eligible titles not identified through the electronic search, as suggested by Greenhalgh and Peacock (15). The Really Simple Syndication tool for PubMed was employed to identify new titles recently indexed and fitted to search strategy. Discrepancies of titles retrieved through the electronic and manual search strategies were resolved by discussion with a third reviewer (DPO). The search details tailored for each database are depicted in supplementary material.

- Inclusion and exclusion criteria

Inclusion criteria: prospective human randomized controlled trials (RCTs), with a split mouth or parallel design, comparing different flap approaches, and evaluating postoperative pain perception by means of a visual analog scale (VAS).

Exclusion criteria: narrative or literature reviews, cross-sectional studies, preclinical and *in vitro* studies, case reports, case series, retrospective studies, posters and conference abstracts, or any study lacking a comparator group.

- Screening and selection of papers

Titles and abstracts were screened by two reviewers (PGS and DSP) on an independent basis. Full-text reports were obtained and reviewed for studies that seemed to meet the inclusion criteria. Kappa scores (Cohen’s ĸ coefficient) were employed during full-text assessment to ensure eligibility and level of agreement between the two reviewers. Disagreements were resolved by discussion and consultation with a third author (MPD). Data referred to the screening process stages are provided in supplementary material.

- Search outcomes and evaluation

Two authors (PGS and DSP) extracted the variables of interest in duplicate, using predefined Excel spreadsheets (Excel for Mac ver. 16.16.2, Microsoft®, Redmond, WA, USA). Disagreements were resolved by discussion with a third reviewer (DPO). In the event of missing data, a request was sent to the authors. Data of included studies were extracted seeking comparability: author, study design, study setting, number of patients, number of teeth (maxilla or mandible), follow-up and surgical technique. The compiled secondary outcomes in turn comprised swelling, trismus and alveolar osteitis.

Primary outcome: Patient postoperative pain perception after third molar extraction, scored by the VAS.

Secondary outcomes: The secondary outcomes were swelling, trismus and alveolar osteitis.

- Risk of bias in individual studies.

Quality assessment was made in duplicate by two independent reviewers (PGS and DSP). The Cochrane risk of bias (RoB) tool was used for the assessment of randomized controlled trials (RCTs). Risk of bias was scored following the recommendations of the Cochrane Handbook for Systematic Reviews of Interventions 5.1.0 (http://handbook.cochrane.org) as follows: low risk of bias (all domains met); high risk of bias (≥ 1 domain not met); and unclear (≥ 1 domain partially met). Reviewer disagreements were discussed to reach consensus.

- Summary of measures and synthesis of results

A meta-analysis was performed to assess pain perception and trismus one week after surgery. A VAS of 0-10 was used for pain assessment. If a study provided results based on a VAS of 0-100, conversion was made seeking comparability. Trismus was measured as interincisal distance in mm or cm. The weighted mean difference (WMD) was the global measure of effect for random-effects meta-analysis. The test is based on z distribution and 95% confidence intervals. Data were represented by means of forest plots, and the relative weight of each meta-analyzed study was estimated. The I2 index of heterogeneity and the corresponding nullity statistical Q-test was calculated, with I2 values of 25%, 50% and 75% being interpreted as reflecting low, moderate and high heterogeneity. Publication bias was investigated by visual detection on the funnel plot, employing the Egger test. In the case of high heterogeneity, a sensitivity and meta-regression analysis was performed to detect the effect of potential confounders upon the results. A level of significance of 5% (α=0.05) is established. The R 3.0.2 statistical package (R Foundation for Statistical Computing, Vienna, Austria) was employed to perform the meta-analysis.

## Results

- Study selection

A total of 1314 potentially eligible titles were identified. After the removal of duplicates and full-text assessment, 11 RCTs were finally included: 8 split-mouth and three parallel trials. No titles were retrieved from other sources (e.g., gray literature and reference lists). The reviewers showed excellent agreement (ĸ = 0.92) during the screening process, according to the Landis and Koch scale (16). A summary of study characteristics is provided in Table 1.

- Risk of bias within studies

**Figure 1:**
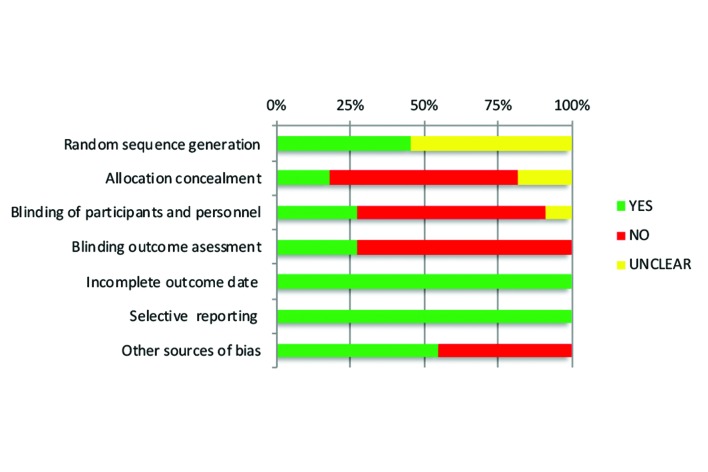
Cochrane´s assessment tool for risk of bias.

The included RCTs showed high to low risk of bias. Only one study was rated as presenting low RoB (17). Allocation concealment, the blinding of participants and outcome assessors were the most critical aspects in the methodological appraisal (Fig. 1).

**Table 1:**
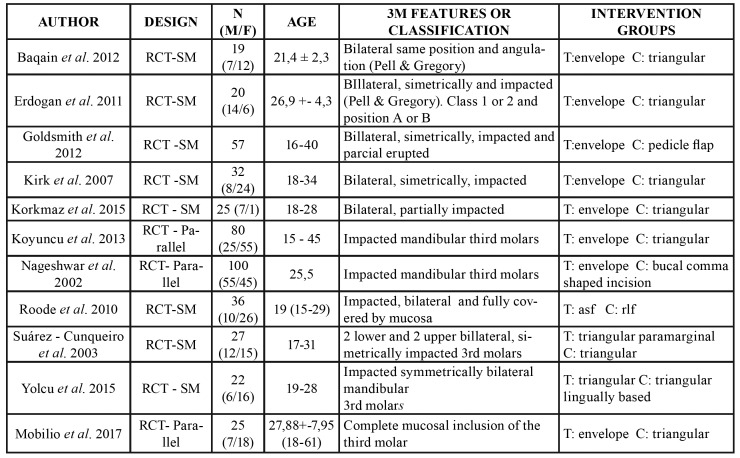
Descriptive summary of included studies.

- Study characteristics

The included studies were published between 2003 and 2017 (Table 1). The 11 articles comprised 443 patients with a total of 661 impacted third molars. Nine studies assessed different incisions with the triangular flap as common comparator. Five evaluated the differences in postoperative pain perception between the triangular flap and the envelope flap (2,7,17-19). Some studies used small variations of the triangular flap and envelope flap, or adopted different flap designs (3,5,6,8,9).

- Qualitative synthesis of the study outcomes

**Figure 2:**
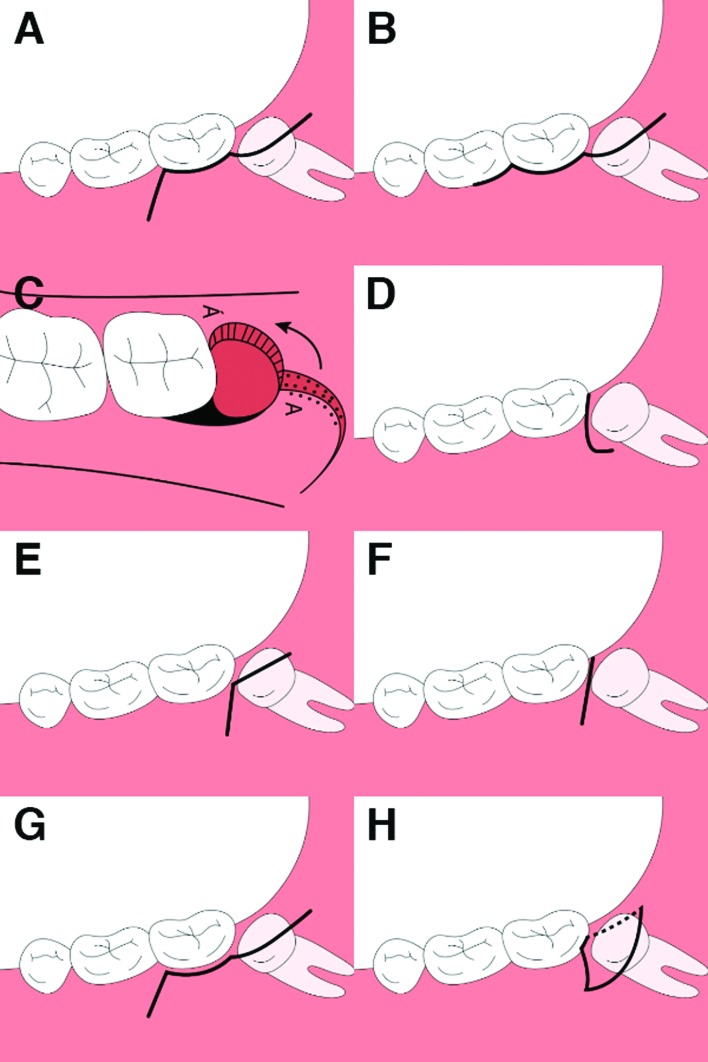
Triangular flap (A); envelope flap (B); pedicle flap (C); comma shpaed incision (D); reverse-L flap "RLF" (E); alternative surgical flap "ASF" (F); paramarginal triangular flap (G); lingually-based triangular flap (H).

Flap designs and pain perception: The primary review outcome was the difference in postoperative pain based on the VAS between patients who underwent triangular flap surgery and those in which other flap designs were used. Regarding the flaps reported in the available literature, the triangular flap began with a distal incision from the mandibular ramus to the distal aspect of the second molar. The incision was continued through the sulcus, and before reaching the papilla between the first and second molars, a buccal releasing incision was made to allow good visibility and access (Fig. 2). The envelope flap in turn was based on a sulcular incision from the first to the second mandibular molar, with a distal releasing incision along the external oblique ridge to the mandibular ramus. Unlike in the case of the triangular flap, there was no buccal releasing incision (Fig. 2). Some authors compared other less common flaps.

Goldsmith *et al*. compared postoperative pain perception between the envelope flap and the pedicle flap using a VAS at 2 and 7 days. The authors recorded slightly higher pain scores ​​in patients operated upon with the pedicle flap (Fig. 2) (5). Nageshwar *et al*. in turn compared the buccal comma-shaped incision versus the envelope flap (Fig. 2). They assessed perceived postoperative pain using a VAS at 1, 3, 7, and 14 days, and recorded slightly lower pain scores in the patients belonging to the buccal comma-shaped incision group (6).

Roode *et al*. compared the reverse L flap (RLF), (Fig. 2), with the alternative surgical flap (ASF), which involves a single incision line (Fig. 2). The ASF group showed lower pain perception scores than the RLF group during the first postoperative week (3). Suárez-Cunqueiro *et al*. introduced the paramarginal triangular flap, which consists of a triangular flap, but performing the incision 2 mm from the free gingival margin (Fig. 2). There were no significant differences between the marginal and paramarginal flaps in terms of perceived pain (9). Yolcu *et al*. in turn proposed a variation of the triangular flap, described as a lingually based triangular flap (Fig. 2). This was a triangular flap, but performing the initial incision in the vestibule, followed by extension to the lingual area (in contrast to what is done in the traditional triangular flap). The lingually based triangular flap yielded higher pain scores at 12 hours postsurgery (*p* < 0.05) (8).

Trismus: Only 7 studies assessed trismus (2,4,6-9,18), which was measured as the interincisal distance during mouth opening. The authors concluded that the triangular flap results in a significantly greater reduction in maximum interincisal mouth opening at 7 and 14 days postsurgery (*p*< 0.05). Nageshwar *et al*. [2002] in turn reported less trismus at days 1 and 3 using a buccal comma-shaped incision versus an envelope flap (6). Suarez-Cunqueiro *et al*. found no significant differences between the marginal and paramarginal flaps in terms of maximum mouth opening (9). No significant differences were found by Yolcu *et al*. between the triangular flap and the lingually based triangular flap (8). Lastly, Mobilio *et al*. recorded greater maximal interincisal opening using the envelope flap versus the triangular flap, but statistical significance was not reached (18).

Swelling: Ten studies evaluated postoperative swelling (2-8,17-19). The lack of a common method for evaluating swelling precluded proper and quantitative comparison among the different studies. Some authors (2,4,18,19) reported more swelling with the triangular flap than with the envelope flap. Other authors (7) reported greater swelling with the envelope flap than with the triangular flap, however (6) observed less swelling with the comma incision flap than when using the envelope flap. Roode and Butow in turn obtained greater swelling values using the RLF flap versus the ASF flap (3). Goldsmith *et al*. recorded more swelling with the pedicle flap than with the envelope flap (5), while Korkmaz, Mollaoglu and Ozmeriç reported greater swelling on day 2 with the three-cornered LRF flap than with the envelope flap (17). Finally, Yolcu and Acar recorded no differences in swelling between the triangular flap and the lingually-based triangular flap (8).

Osteitis: Four studies reported this outcome. Two articles described a higher prevalence of osteitis in patients treated with an envelope flap versus pedicle and triangular flaps (4,5). However, Koyuncu *et al*. [2013] reported more cases of osteitis in patients treated with triangular flaps than in patients treated with envelope flaps (7). Mobilio *et al*. [2017] reported a greater incidence of osteitis with the triangular flap than with the triangular lingually-based flap (18).

- Quantitative synthesis - meta-analysis

Data from 5 studies comprising 166 patients (232 teeth) were included in the present meta-analyses. Two analyses were performed according to the outcome of interest. All 5 studies were considered for postoperative pain perception (2,4,7,17,19). However, only two studies were included in the trismus meta-analysis (7,19).

**Figure 3:**
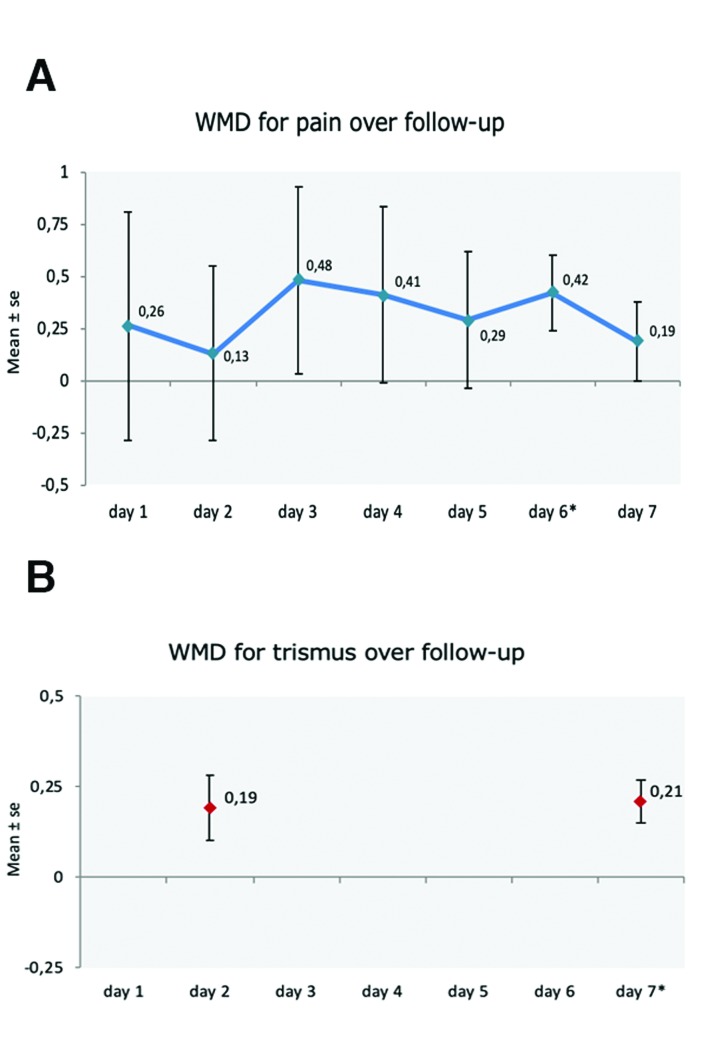
Cummulative plot of random effects meta-analyses a week postoperative. Pain (A); Trismus (B).

Pain perception: A descriptive summary of the data included in the analysis corresponding to the first postoperative week is provided in Table 2. No statistically significant differences were found between the envelope flap and the triangular flap, except on the sixth day (Fig. 3). Lesser pain perception was recorded in the control group (triangular flap), with a weighted mean difference (WMD) of 0.42 (95%CI: 0.06-0.77) (*p* = 0.021). No inconsistency was observed: I2 = 0%. The WMD corresponding to pain perception is shown in Fig. 3.

**Table 2:**
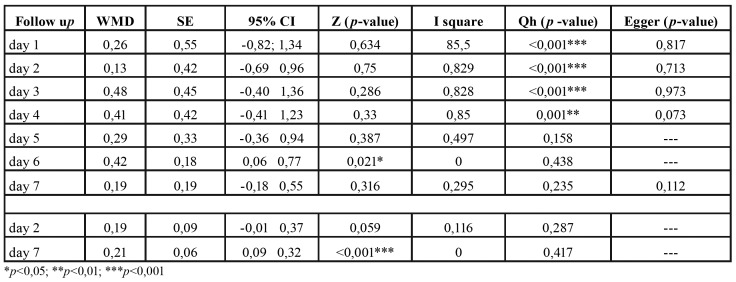
Pain and trismus meta-analyses data.

Trismus: Only two studies (144 teeth) were subjected to meta-analysis (4,7), comparing mouth opening after 2 and 7 days. On day 2, no significant differences were found between the test group and the control group, though mouth opening was greater with the envelope flap than with the triangular flap. However, on day 7, mouth opening was significantly greater in the envelope flap group, with a WMD of 0.21 (95%CI: 0.09-0.32) (*p* = 0.001). No inconsistency was observed: I2 = 0%. The WMD corresponding to trismus between the test and control groups is shown in Fig. 3.

## Discussion

The main objective of this study was to determine the influence on patient morbidity of the different flaps used in impacted third molar extraction. The most widely used flaps were the triangular and the envelope designs, though some authors proposed variations of these designs, or other flaps. The triangular flap was taken as the comparator design in the 11 included studies.

The present systematic review found postoperative pain to decrease continuously over time in all of the studies. No significant differences were found between the triangular flap and the envelope flap, except on the sixth day after surgery, when the triangular flap yielded lower pain perception values than the envelope flap (*p* = 0.021). On the other postoperative days the triangular flap also showed slightly lower pain values, but without reaching statistical significance. All the studies showed similar pain perception scores. Some authors (17) reported higher pain perception values in the triangular flap group during the first two days, though from the third postoperative day higher pain perception values were recorded in the envelope flap group. According to one study (7), the triangular flap causes less pain throughout the first postoperative week. However, according to other authors who analyzed pain on the third and seventh postoperative days (19), the triangular flap causes comparatively more pain. The triangular flap was found to be slightly less painful than the envelope flap (non significant difference). Likewise, Coulthard *P* *et al*. [2014], reported that a small reduction in the mean pain score at 24 hours is expected when a triangular flap is used (20). A logical explanation for this could be that this flap design allows better vision and better access to the impacted third molar area, which in turn makes the surgical procedure easier and faster. Regarding other flaps studied, the pedicle flap was associated to lower pain perception scores ​​than the envelope flap (5). The incidence of osteitis was higher with the envelope flap (5 cases) than with the pedicle flap (0 cases).

Regarding trismus outcomes, the findings suggest significantly greater mouth opening on the seventh postoperative day in the envelope flap group than in the triangular flap group (*p* = 0.001). According to Chen *et al*. (1), the envelope flap seems to be associated with slightly greater loss of clinical attachment level (CAL) distal to the second molar. According to Coulthard *P* *et al*. (20), triangular flap have reduced risk of alveolar osteitis, even though Koyuncu *et al*. [2013] reported more cases of osteitis in patients treated with triangular flaps than in patients treated with envelope flaps (7).

The strengths of this systematic review are the comprehensive electronic literature search and the efforts to use methodology tools for qualitative and quantitative synthesis of the data. Also, the screening process and summary of judgments to reach the risk of bias assessment are described transparently in the supplementary material. Nevertheless, some limitations also should be mentioned, such as the methodological issues found in the included studies, and which are characteristic of clinical trials in dentistry (21,22). The statistical heterogeneity observed was a consequence of both clinical and methodological heterogeneity among the included studies. In this regard, the intervention effect sizes were influenced by different parameters: patients characteristics (e.g., sex and age), intervention features (e.g., surgeon experience, surgical materials, duration of surgery), measurement of outcomes (e.g., VAS, swelling assessment methods, outcomes follow-up) and methodological factors (risk of bias for allocation concealment, lack of blinding of participants and outcome assessors, and other sources of bias in RCTs). In addition, mention must be made of the impossibility of including more studies because they were not RCTs or did not assess pain perception based on a VAS. On the other hand, the VAS is a subjective scale, and there is evidence that it could be influenced by a lack of participants and by personnel blinding (21).

Although it has been shown that on the seventh postoperative day the degree of trismus is greater in the envelope flap group, the difference is small. In any case, we believe that in the final balance of advantages, the triangular flap is superior to the envelope flap. In future research, it would be advisable to conduct controlled and blinded studies as far as possible. It is necessary to standardize a method as objective as possible in order to assess swelling and be able to compare it between different flap designs. Furthermore, we included mostly small randomized trials and observed methodological flaws that may influence the meta-analytical results across studies. Studies should be made with sample size calculation, and we recommend the inclusion of new flap types, in order to compare them with the triangular flap and not only with the envelope flap. From our point of view, further research is needed, comparing alternative flaps and the triangular flap, and also increasing the sample size.

## Conclusions

Despite its limitations, the present meta-analysis found no clear effect of the flap design in patient morbidity. No clinically relevant differences were found regarding pain perception and trismus when comparing triangular and envelope flaps. Further research is needed to determine if the flap design increases the rate of alveolar osteitis.

## References

[1] Chen YW, Lee CT, Hum L, Chuang SK (2017). Effect of flap design on periodontal healing after impacted third molar extraction: a systematic review and meta-analysis. Int J Oral Maxillofac Surg.

[2] Baqain ZH, Al-Shafii A, Hamdan AA, Sawair FA (2012). Flap design and mandibular third molar surgery: A split mouth randomized clinical study. Int J Oral Maxillofac Surg.

[3] Roode GJ, Butow K (2010). An alternative surgical flap design for impacted third molars: a comparison of two different surgical techniques. SADJ.

[4] Kirk DG, Liston PN, Tong DC, Love RM (2007). Influence of two different flap designs on incidence of pain, swelling, trismus, and alveolar osteitis in the week following third molar surgery. Oral Surg Oral Med Oral Pathol Oral Radiol.

[5] Goldsmith SM, Silva RK De, Tong DC, Love RM (2012). Influence of a pedicle flap design on acute postoperative sequelae after lower third molar removal. Int J Oral Maxillofac Surg.

[6] Nageshwar (2002). Comma incision for impacted mandibular third molars. Int J Oral Maxillofac Surg.

[7] Koyuncu BÖ, Çetingül E (2013). Short-term clinical outcomes of two different flap techniques in impacted mandibular third molar surgery. Oral Surg Oral Med Oral Pathol Oral Radiol.

[8] Yolcu U, Acar AH (2015). Comparison of a new flap design with the routinely used triangular flap design in third molar surgery. Int J Oral Maxillofac Surg.

[9] Suarez-Cunqueiro MM, Gutwald R, Reichman J, Otero-Cepeda XL, Schmelzeisen R (2003). Marginal flap versus paramarginal flap in impacted third molar surgery: A prospective study. Oral Surg Oral Med Oral Pathol Oral Radiol Endod.

[10] Guyatt GH, Sackett DL, Cook DJ (1993). Users' Guides to the Medical Literature: II. How to Use an Article About Therapy or Prevention A. Are the Results of the Study Valid? JAMA.

[11] Walter SD, Sun X, Heels-Ansdell D, Guyatt G (2012). Treatment effects on patient-important outcomes can be small, even with large effects on surrogate markers. J Clin Epidemiol.

[12] Liberati A, Altman DG, Tetzlaff J, Mulrow C, Gøtzsche PC, Ioannidis JPA (2009). The PRISMA Statement for Reporting Systematic Reviews and Meta-Analyses of Studies That Evaluate Health Care Interventions: Explanation and Elaboration. PLoS Med.

[13] Shea BJ, Reeves BC, Wells G, Thuku M, Hamel C, Moran J (2017). AMSTAR 2: a critical appraisal tool for systematic reviews that include randomised or non-randomised studies of healthcare interventions, or both. BMJ.

[14] Stone PW (2002). Popping the (PICO) question in research and evidence-based practice. Appl Nurs Res.

[15] Greenhalgh T, Peacock R (2005). Effectiveness and efficiency of search methods in systematic reviews of complex evidence: audit of primary sources. BMJ.

[16] Landis JR, Koch GG (1977). The measurement of observer agreement for categorical data. Biometrics.

[17] Korkmaz YT, Mollaoglu N, Ozmeriç N (2015). Does laterally rotated flap design influence the short-term periodontal status of second molars and postoperative discomfort after partially impacted third molar surgery?. Int J Oral Maxillofac Surg.

[18] Mobilio N, Vecchiatini R, Vasquez M, Calura G, Catapano S (2017). Effect of flap design and duration of surgery on acute postoperative symptoms and signs after extraction of lower third molars: A randomized prospective study. J Dent Res Dent Clin Dent Prospects.

[19] Erdogan Ö, Tatlı U, Üstün Y, Damlar I (2011). Influence of two different flap designs on the sequelae of mandibular third molar surgery. Oral Maxillofac Surg.

[20] Coulthard P, Bailey E, Esposito M, Furness S, Renton TF, Worthington HV (2014). Surgical techniques for the removal of mandibular wisdom teeth. Cochrane Database Syst Rev.

[21] Saltaji H, Armijo-Olivo S, Cummings GG, Amin M, da Costa BR, Flores-Mir C (2018). Impact of Selection Bias on Treatment Effect Size Estimates in Randomized Trials of Oral Health Interventions: A Meta-epidemiological Study. J Dent Res.

[22] Saltaji H, Armijo-Olivo S, Cummings GG, Amin M, Flores-Mir C (2017). Randomized clinical trials in dentistry: Risks of bias, risks of random errors, reporting quality, and methodologic quality over the years 1955-2013. PLoS One.

